# A Comparative Study on Surgical Treatment of Valvular Heart Disease between High-Volume Cardiac Centers in China and STS Data

**DOI:** 10.3390/jcdd9070212

**Published:** 2022-07-02

**Authors:** Hongyuan Lin, Jiamiao Gong, Yongjian Wu, Zhe Zheng, Jianfeng Hou

**Affiliations:** Cardiac Surgery Center, Fuwai Hospital, Chinese Academy of Medical Sciences and Peking Union Medical College, Beijing 100037, China; linhongyuan@fuwai.com (H.L.); gongjiamiao@fuwai.com (J.G.); wuyongjian@fuwai.com (Y.W.); zhengzhe@fuwai.com (Z.Z.)

**Keywords:** valvular heart disease, VHD, surgery, quality, CCSR, STS

## Abstract

The goal of this study is to summarize valvular surgery data from the Chinese Cardiac Surgery Registry (CCSR) and compare it to the most recent data from the Society of Thoracic Surgeons (STS). From 2016 to 2018, a total of 34,386 cases of the seven most common valvular surgical procedures was obtained from the CCSR. We calculated the proportions of different procedures in the CCSR cohort (n = 34,386) as well as the change in operation volume for each procedure. We also compiled rates of postoperative in-hospital mortality and five major complications across all procedures. All of the results were compared to the STS data. The CCSR and STS data showed divergent trends in valvular heart disease features and operation volume. Although the proportion of MV repair in the CCSR (13.7%) data was lower than in the STS data (23.2%), it demonstrated a substantial upward trend. In terms of operation volume, the CCSR data showed an upward trend, but the STS data showed a downward trend. CCSR procedures showed lower mortality (2% vs. 2.6%), reoperation (2.8% vs. 4.3%), and permanent stroke (0.5% vs. 1.6%) rates than STS procedures but higher rates of prolonged ventilation (22.4% vs. 10.4%) and renal failure (5.6% vs. 3.2%). Valvular surgery quality in China’s leading cardiac hospitals is roughly comparable to that in the United States. China, on the other hand, has some shortcomings that need improvement.

## 1. Introduction

The mortality rate of valvular heart disease (VHD) has decreased over time [1,2], owing to greater health care coverage and enhanced cardiac medical technology [1]. The characteristics of VHD have changed dramatically as a result of prolonged survival and an aging population [3]. The Society of Thoracic Surgeons (STS) released an update on the outcomes and quality of adult cardiac surgery in 2021, revealing significant variations from ten years prior [4]. For cardiac surgeons throughout the world, the persisting burden [4,5] and the current scenario of VHD treatment pose significant challenges. In China, however, there is no nationwide study on the surgical outcomes and quality of VHD. By reviewing and summarizing VHD data from the Chinese Cardiac Surgery Registry (CCSR) [6] and comparing it to updated data [4] from the STS database, we hoped to gain a better understanding of the surgical treatment of VHD in China.

## 2. Materials and Methods

### 2.1. Data Source

A steering committee comprised of cardiac surgeons and researchers from Fuwai Hospital and the National Center for Cardiovascular Diseases (NCCD) oversees the registry (CCSR). This database contains information on VHD surgery from 94 hospitals. Each participating hospital performs more than 100 cardiac surgeries per year and is required to record cases using a standardized case report form (CRF). These are the leading cardiac centers, and they share many features with other large cardiac care centers in China. According to the Chinese Society of Extracorporeal Circulation’s annual surveys, we estimate that our database contains approximately 30% to 40% of all valve operations and represents performance in large cardiac centers [6].

### 2.2. Patients

We identified 43,877 patients in the CCSR database who underwent valvular surgery between 1 January 2016 and 31 December 2018. Among these patients, we excluded a total of 5746 patients who were under the age of 18 and 3745 patients who underwent surgery beyond the seven most common procedures, including aortic valve replacement (AVR), mitral valve replacement (MVR), mitral valve repair (MV repair), AVR concomitant with coronary artery bypass grafting (CABG), MVR concomitant with CABG, MV repair concomitant with CABG, and AVR concomitant with MVR. A total of 34,386 cases of the seven most common valve surgical procedures was collected from the CCSR database for analysis. 

### 2.3. Statistical Analysis

We calculated the proportion of each procedure. In order to understand the trends in VHD surgery, the change of each procedure volume from 2016 to 2018 in the CCSR cohort was compared with the change of the same procedure volume from 2018 to 2019 in the STS data. We selected 6 perioperative outcomes on which we performed the comparison between the CCSR data and STS data. The 6 outcomes included operative in-hospital mortality, reoperation, deep sternal wound infection (DSWI) or mediastinitis, permanent stroke, prolonged ventilation (>24 h), and renal failure. These 6 outcomes were also found in the STS database [7] and are defined as follows: (1) operative mortality, defined as all deaths, regardless of cause, occurring during the hospitalization in which the operation was performed, even if after 30 days (includes patients transferred to other acute care facilities), and all deaths, regardless of cause, occurring after discharge from the hospital but before the end of the 30th postoperative day; (2) reoperation for bleeding, tamponade, or any cardiac reason; (3) deep sternal wound infection (DSWI) or mediastinitis occurring during the index hospitalization or within 30 days of operation; (4) permanent stroke—an acute episode of focal or global neurologic dysfunction caused by brain, spinal cord, or retinal vascular injury as a result of hemorrhage or infarction in which the neurologic dysfunction lasts for more than 24 h; (5) prolonged ventilation—more than 24 h; (6) renal failure—a new requirement for dialysis or meeting the RIFLE (Risk, Injury, Failure, Loss of kidney function, and End-stage kidney disease) criteria based on creatinine levels or glomerular filtration rate [8]. The incidence rates of these 6 outcomes for each procedure in the CCSR cohort were compared with the corresponding rates reported in the STS update. Categorical variables were reported as frequencies (percentages) and compared between groups by chi-square test. A *p*-value of less than 0.05 was considered statistically significant. R software version 4.0 was used for statistical analyses. R software version 4.0 and GraphPad Prism for Windows version 6.0 were used to create graphs.

## 3. Results

This section is divided by subheadings. It should provide a concise and precise description of the experimental results, their interpretation, as well as the experimental conclusions that can be drawn.

### 3.1. Proportions of More Commonly Performed VHD Surgeries

**Figure 1:**
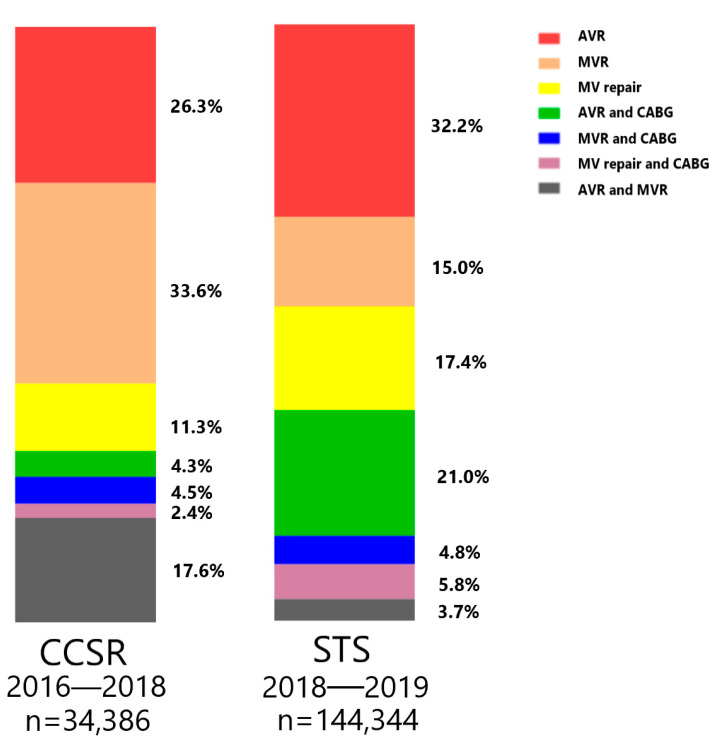
Proportions of the seven most commonly performed VHD surgeries. CCSR: Chinese Cardiac Surgery Registry; STS: Society of Thoracic Surgeons; AVR: aortic valve replacement; MVR: mitral valve replacement; CABG: coronary artery bypass grafting.

Figure 1 displays the proportions of the seven most commonly performed VHD surgeries in the CCSR database from 2016 to 2018 (n = 34,386) and the proportions of the same VHD surgeries in the STS database from 2018 to 2019 (n = 144,344). It can be disclosed that the proportion of mitral valve surgery in the CCSR data (69.4%) was significantly larger than that in the STS data (46.7%); however, fewer MV repairs were found in the CCSR data (13.7%) than in the STS data (23.2%). There were more concomitant CABGs in the STS data (31.6%) than in the CCSR data (11.2%).

### 3.2. Trends in VHD Surgical Procedures

Table 1 shows the change in volume of each procedure in the CCSR data from 2016 to 2018 and in the STS data from 2018 to 2019. We could see in the CCSR data that the overall volume of VHD surgery increased steadily (1.6%); the volumes of MV repair (16.1%) and AVR (11.9%) increased the most. However, the volumes of AVR plus MVR (−13.1%) and isolated MVR (−4.1%) decreased. On the contrary, in the STS data, the overall surgery volume showed a downward trend (−9.1%), and the most obvious decline was in AVR with and without CABG (−11.5% and −18.3%). It is worth noting that the concomitant CABG procedures increased by 8% from 2016 to 2018 in the CCSR data. 

### 3.3. Distribution of In-Hospital Mortality and Other Major Complications Rates in Different Procedures

**Figure 2:**
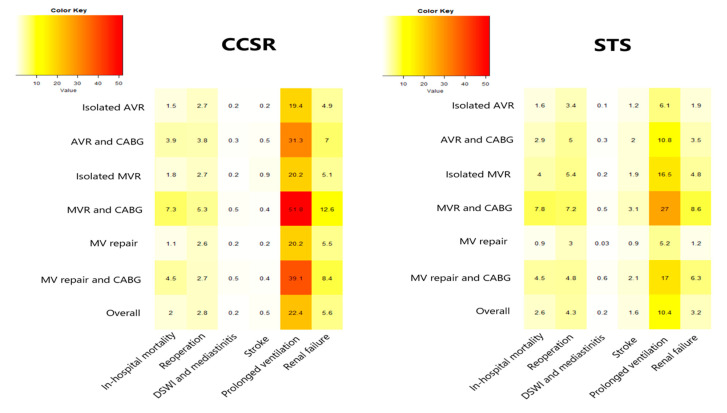
Distribution of in-hospital mortality and other major complication rates (%) in different procedures. CCSR: Chinese Cardiac Surgery Registry; STS: Society of Thoracic Surgeons; AVR: aortic valve replacement; MVR: mitral valve replacement; CABG: coronary artery bypass grafting; DSWI: deep stern wound infection.

Figure 2 shows a comparison of in-hospital mortality and other major complication rates in different procedures between the CCSR data (2016–2018) and the STS data (2019). We found that the distribution of in-hospital mortality and other major complication rates in different procedures showed a similar pattern in the CCSR and STS data. VHD surgery concomitant with CABG indicated a poorer outcome in both CCSR and STS data (the darker the color, the larger the rate). Of all the seven procedures, MVR plus CABG presented the highest mortality (7.3% in CCSR data and 7.8% in STS data) and complication rates.

### 3.4. Comparison of Outcomes in the Whole Cohort between CCSR and STS data

**Figure 3:**
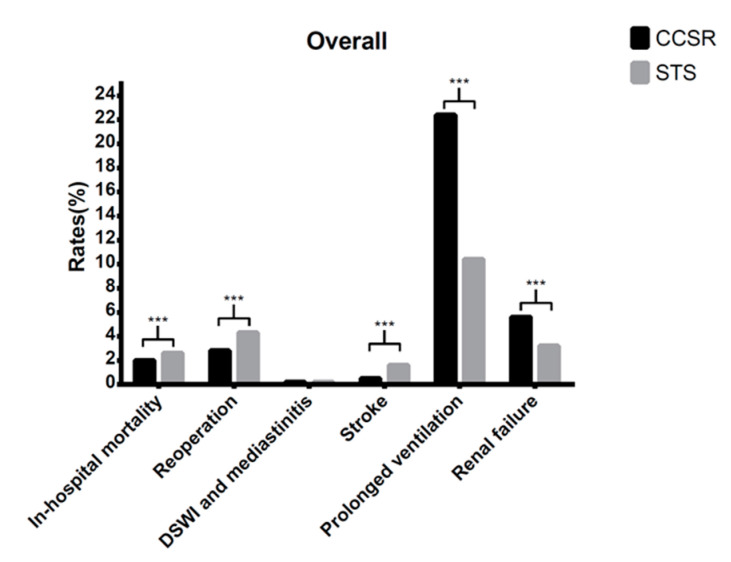
Comparison of outcomes in the whole cohort between CCSR and STS procedures. CCSR: Chinese Cardiac Surgery Registry; STS: Society of Thoracic Surgeons; DSWI: deep sternal wound infection. ***: *p*-value < 0.001.

Figure 3 shows the comparison of in-hospital mortality and complication rates in all seven VHD procedures between CCSR and STS data. Overall, CCSR procedures was superior to STS procedures in terms of in-hospital mortality (2% vs. 2.6%), reoperation (2.8% vs. 4.3%), and stroke (0.5% vs. 1.6%). However, compared with STS procedures, CCSR procedures had significantly higher rates of prolonged ventilation (22.4% vs. 10.4%) and renal failure (5.6% vs. 3.2%).

### 3.5. Comparison of Outcomes in Different Procedures between CCSR and STS data

**Figure 4:**
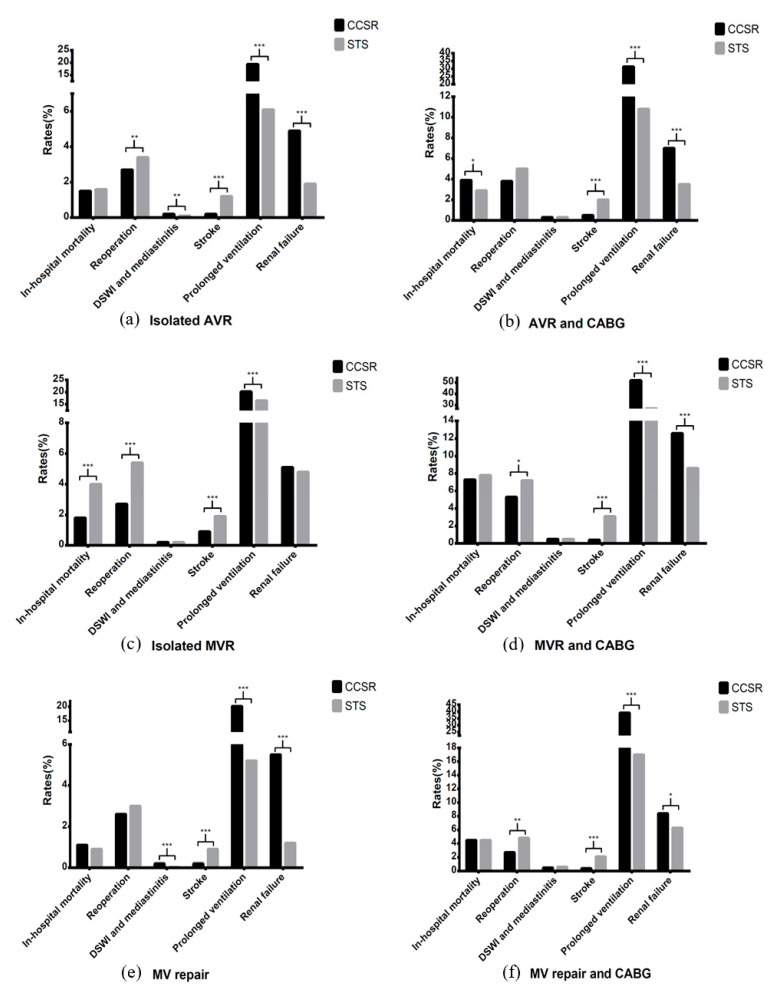
Comparison of outcomes in different procedures between CCSR and STS data. CCSR: Chinese Cardiac Surgery Registry; STS: Society of Thoracic Surgeons; AVR: aortic valve replacement; MVR: mitral valve replacement; CABG: coronary artery bypass grafting; DSWI: deep sternal wound infection. *: 0.01 < *p*-value < 0.05; **: 0.001 < *p*-value < 0.01; ***: *p*-value < 0.001.

Figure 4a–f show the comparison of in-hospital mortality and complication rates in different procedures between CCSR and STS data.

## 4. Discussion

VHD surgery is one of the most common cardiac operations. The quality of VHD surgery indicates the level of cardiac surgery in a country to some extent. Quality control studies are critical for improving the surgical treatment of VHD. Unlike coronary artery disease (CAD), however, VHD has multifarious features and can be treated with a variety of surgical methods. Globally, only some developed countries in Europe and North America have conducted nationwide research [4,9] based on national databases on quality controls in VHD surgery. We chose seven of the most commonly performed VHD procedures from the CCSR database for investigation in order to reveal the state and problems of VHD surgery in China. We attempted to find drawbacks in VHD surgical treatment by comparing it to STS data and to provide references for improving medical care in China.

The CCSR [6], which is similar to the STS in the United States, provides a comprehensive and scientifically rigorous approach to objectively assessing quality and measuring performance. A committee of Chinese cardiac surgeons and researchers from universities and major hospitals oversees the registry, which aims to evaluate surgical outcomes in patients undergoing cardiac surgery. Consequently, CCSR data is representative and generalizable in the field of cardiac surgery in China.

The mitral valve remains the most common surgical site for VHD in CCSR data (accounted for 69.4% of all VHD surgeries). Compared with STS data, the proportion of MV repair in the CCSR data was significantly lower (only 13.7%). The main reason is that rheumatic heart disease (RHD) is still the first major cause of moderate or severe VHD in China [10,11,12,13], especially among the mitral valve lesions. Although the superiority of valve repair over replacement in degenerative mitral valve disease has been well established, its role in RHD has remained controversial. The repaired rheumatic mitral valve is frequently thought to have inferior durability due to the ongoing inflammatory process and the resulting risk of failure and reoperation as a result of its notoriously complicated pathology [14,15]. Dibardino DJ et al. [14] summarized 40 years of experience in MV repair and the 30-year follow-up results of 1503 MV repair cases, showing that in degenerative cases, freedom from reoperation at 10 and 20 years was 90% and 82%, respectively, which were significantly better rates than for rheumatic disease (66% and 34%, *p* < 0.001). Furthermore, the complexity of RHD frequently requires numerous repair techniques to be used concurrently, demanding the proficiency of high-volume specialist surgeons [16]. Due to the technical complexity of MV repair for RHD, limited expertise in MV repair for RHD, and concerns about durability, most surgeons currently replace over 90% of rheumatic mitral valves [17]. However, from 2016 to 2018, MV repair was the fastest-growing operation in the CCSR data (Table 1). This is closely associated with recent changes in the VHD spectrum in China: the prevalence of degenerative VHD is soaring, owing to longer survival and an aging population [11,12,13]. Another issue with an aging population is the occurrence of concomitant chronic conditions, such as CAD. In the CCSR data, the number of concomitant CABG surgeries increased by 8% from 2016 to 2018, resulting in an increasing demand for Chinese cardiac surgeons.

In the STS data, VHD surgery generally showed a downward trend from 2018 to 2019 (Table 1), and AVR showed the largest decline, which was mainly attributed to the vigorous development of transcatheter AVRs (TAVRs) in recent years. Carroll, J.D. [18] reported the data of the STS–ACC TVT Registry (Society of Thoracic Surgeons–American College of Cardiology Transcatheter Valve Therapy Registry) from 2011 to 2019, showing that 276,316 patients had undergone TAVRs in all U.S. states. Volumes have increased every year, exceeding surgical aortic valve replacements in 2019 (72,991 vs. 57,626). Unlike the STS data, the number of VHD surgeries is still increasing in the CCSR data, as is AVR. TAVR is significantly less common in China than it is in the United States for technical and economic reasons. Despite the fact that no nationwide data on TAVRs has been published in China, the largest multi-center retrospective study [19], covering seven major sites in China, found that only 1204 patients have had TAVRs between April 2012 and November 2020 in these seven prominent cardiac centers.

A comparison of surgical outcomes revealed that CCSR procedures outperformed STS procedures in terms of postoperative mortality, postoperative stroke, and reoperation rate, indicating that VHD surgical technology in Chinese large cardiac centers has advanced significantly in recent years, and surgical quality is roughly in line with that of some Western high-income countries. In China, however, there are still obvious flaws in surgical technique details and perioperative management. For example, the rate of prolonged ventilation was much greater than with the STS, implying that some technology and concepts of VHD management in China fall behind those in the United States. Additionally, the rate of postoperative renal failure was much higher in the CCSR data than in the STS data, implying that there is still a lot of room and potential to improve perioperative organ perfusion and protection. Additionally, concomitant CABG was associated with greater rates of in-hospital mortality and other major complications in both CCSR and STS cohorts, confirming that CAD raises the risk of VHD surgery.

### Limitations

In this study, we could only use publicly available STS data, so we were unable to conduct further etiological analyses that needed more detailed data, for example, demographics. Risk-adjusted outcomes could not be made due to the lack of demographic data. Therefore, the comparative analysis could only be based on crude outcomes. In addition, the two groups compared in this study are not in the same time span, and the CCSR data only contain the data of high-level cardiac centers in China and do not cover all cardiac centers in the country, like the STS data.

## 5. Conclusions

Valvular surgery quality in China’s leading cardiac hospitals is roughly comparable to that in the United States. China, on the other hand, has some shortcomings and needs improvement in terms of surgical technical details and complication avoidance.

## Figures and Tables

**Table 1:** Change in procedure volume in CCSR data from 2016 to 2018 and in STS data from 2018 to 2019.

Procedure	CCSR 2016	CCSR 2018	CCSR Change: 2016 to 2018 (%)	STS 2018	STS 2019	STS Change: 2018 to 2019 (%)
Overall	11,850	12,044	1.6 ↑	75,597	68,747	−9.1 ↓
Isolated AVR	3033	3395	11.9 ↑	25,646	20,965	−18.3 ↓
Isolated MVR	4042	3876	−4.1 ↓	10,823	10,748	−0.7 ↓
MV repair	1231	1429	16.1 ↑	12,608	12,570	−0.3 ↓
AVR and CABG	493	547	11.0 ↑	16,100	12,246	−11.5 ↓
MVR and CABG	486	512	5.3 ↑	3554	3441	−3.2 ↓
MV repair and CABG	271	291	7.4 ↑	4151	4153	0.0
AVR and MVR	2294	1994	−13.1 ↓	2715	2624	−3.4 ↓

CCSR: Chinese Cardiac Surgery Registry; STS: Society of Thoracic Surgeons; AVR: aortic valve replacement; MVR: mitral valve replacement; CABG: coronary artery bypass grafting. ↑: increase; ↓: decrease.

## Data Availability

The data presented in this study are available upon request from the corresponding author. The data are not publicly available due to the No.717 Chinese Decree of the State Council, suggesting that the raw data of large samples cannot be shared globally.
